# Beyond the Antioxidant Activity of Dietary Polyphenols in Cancer: the Modulation of Estrogen Receptors (ERs) Signaling

**DOI:** 10.3390/ijms19092624

**Published:** 2018-09-05

**Authors:** Manuela Cipolletti, Virginia Solar Fernandez, Emiliano Montalesi, Maria Marino, Marco Fiocchetti

**Affiliations:** Department of Science, University Roma Tre, Viale Guglielmo Marconi 446, I-00146 Roma, Italy; manuela.cipolletti@uniroma3.it (M.C.); Virginia.solarfernandez@uniroma3.it (V.S.F.); emi.montalesi@stud.uniroma3.it (E.M.)

**Keywords:** polyphenols, cancer, estrogen receptors

## Abstract

The potential “health benefits” of dietary polyphenols have been ascribed to their direct antioxidant activity and their impact on the regulation of cell and tissue redox balance. However, because of the relative poor bioavailability of many of these compounds, their effects could not be easily explained by the antioxidant action, which may occur only at high circulating and tissue concentrations. Therefore, many efforts have been put forward to clarify the molecular mechanisms underlining the biological effect of polyphenols in physiological and pathological conditions. Polyphenols’ bioavailability, metabolism, and their effects on enzyme, membrane, and/or nuclear receptors and intracellular transduction mechanisms may define the overall impact of these compounds on cancer risk and progression, which is still debated and not yet clarified. Polyphenols are able to bind to estrogen receptor α (ERα) and β (ERβ), and therefore induce biological effects in human cells through mimicking or inhibiting the action of endogenous estrogens, even at low concentrations. In this work, the role and effects of food-contained polyphenols in hormone-related cancers will be reviewed, mainly focusing on the different polyphenols’ mechanisms of action with particular attention on their estrogen receptor-based effects, and on the consequences of such processes on tumor progression and development.

## 1. Introduction

Polyphenols constitute one of the most numerous and widely distributed groups of natural products in the plant kingdom. They represent the secondary metabolites that are essential for plant physiology, as they are involved in several functions including pigmentation, pollination, the inhibition of pathogen development, and protection against UltraViolet (UV) radiation, photosynthetic stress, reactive oxygen species, wounds, and herbivores [1].

Polyphenols are present in a large variety of edible plants that are consumed daily in the human diet as food (i.e., vegetables, cereals, legumes, fruits, nuts, etc.) and beverages (i.e., wine, cider, beer, tea, cocoa, etc.) [2]. Growing interest about these dietary polyphenols derives from epidemiological studies that support a positive relationship between a high dietary intake of polyphenols and the reduced risk of several chronic diseases in human beings, including neurodegenerative diseases, cardiovascular diseases, and specific type of cancers [3,4,5], although contrasting data have been reported about the correlation between the consumption of dietary polyphenols and cancer risk [6].

The high number of different polyphenols that are found in food, as well as in mixture, and the extensive metabolism that polyphenols undergo in human gut and liver as well as the several, and somewhat contradictory, action mechanisms reported have been the major difficulties in elucidating the effects of dietary polyphenols on cancer [3]. Indeed, for years, the direct and indirect antioxidant properties of polyphenols have been considered the main, if not the only, action mechanism of dietary polyphenols, on which their “health promoting” effects rely. However, it is now clear that the actions of polyphenols go beyond the regulation of redox balance [4]. In particular, the phenolic ring that characterizes these compounds allows them to bind to estrogen receptor subtypes (i.e., ERα and ERβ) [7,8], inducing estrogenic or antiestrogenic responses in the target cells by blocking or altering the effects of the endogenous hormone (i.e., 17β-estradiol, E2). This evidence led to the insertion of dietary polyphenols in the class of selective ER modulators [9,10], thus rendering necessary the re-evaluation of the effect of polyphenols on cancer cells.

Here, the overview of polyphenols’ chemical structure, sources, and effect on cancer disease will be reviewed, with a particular attention to the effects of polyphenols as ER modulators and the consequences of such function on tumor progression and development.

## 2. Dietary Polyphenols Structure and Source

**Figure 1:**
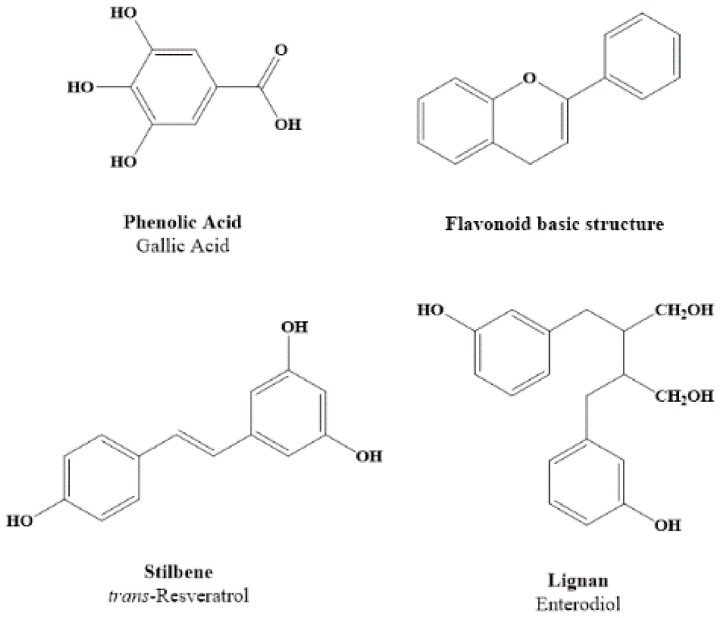
Chemical structure of different groups of polyphenols.

Polyphenols derive from a common intermediate, phenylalanine, or a close precursor, shikimic acid. They arise biogenetically from two main synthetic pathways: the shikimate pathway and the acetate pathway [11]. Natural polyphenols can range from simple molecules, such as phenolic acids, to highly polymerized compounds, such as tannins. Based on their chemical structure, they are divided into several subclasses, such as phenolic acids, flavonoids (flavones, flavonols, flavanones, isoflavonoids, flavanols, anthocyanins), stilbenes, and lignans [12]. Figure 1 illustrates different groups of polyphenols and their chemical structures. All of them contain one or several aromatic rings with at least one hydroxyl group [13]. The polyphenols in plants could be conjugated with one or more sugar residues linked to hydroxyl groups, but direct linkages of the sugar to an aromatic carbon also exist. Linkages with other compounds, such as amines, carboxylic and organic acids, and lipids are common, as are associations with other phenols [1]. Certain polyphenols, such as the flavonol quercetin, are found in all plant products (i.e., fruit, vegetables, cereals, leguminous plants, tea, and wine), but others are specific components of particular foods (i.e., flavanones in citrus fruit, isoflavones in soya, phloridzin in apples). In most cases, food contains complex mixtures of polyphenols, and for many products, the composition of polyphenols is less known (for review, see [14] and the literature cited therein). For instance, apples represent one of the few cases for which the precise composition of the polyphenols is available. Certain red varieties contain in their skin flavanol monomers or oligomers, chlorogenic acid and small quantities of other hydroxycinnamic acids, two glycosides of phloretin, several quercetin glycosides, and anthocyanins such as cyanidin 3-galactoside. The profile of polyphenols for all varieties of apples is practically identical, but the concentrations may significantly differ from 0.1 g of total polyphenols/kg fresh weight to 10g/kg in certain varieties of cider apples [14]. One beverage characterized by a complex mixture of polyphenols is wine. Wine contains flavonols, flavanol, proanthocyanidins, anthocyanins, phenolic acids, hydroxycinnamates, and stilbenes. One of the most studied of these compounds is *trans*-resveratrol, because it is considered the main compound responsible for the benefits of wine on human health, but its concentration varies considerably depending on the vine variety [15].

Polyphenol content in food is also influenced by environmental factors including climatic or agronomic parameters, and, in particular, by the timing of plant light exposure. Moreover, the degree of ripeness affects the concentration of polyphenols. As an example, phenolic acid concentrations decrease, whereas anthocyanins concentrations increase during ripening. Another important factor that affects the content of polyphenols in food is the storage. Indeed, polyphenols are easily oxidized, leading to the formation of polymerized substances, which modify the fruits’ color and organoleptic characteristics. However, cold storage does not affect polyphenols content [16,17]. The amount of polyphenols in foods might be also influenced by the methods of food preparation; the peeling of fruits and vegetables can significantly reduce polyphenol content, because they are often present in high concentrations in the outer parts. In addition, onions and tomatoes lose about 75% of their initial quercetin content after boiling for 15 min, 65% after cooking in a microwave oven, and 30% after frying [18].

### 2.1. Dietary Polyphenols’ Bioavailability and Metabolites

Bioavailability is a term used to indicate the proportion of the nutrient that is digested, adsorbed, and metabolized through normal pathways. Consequently, to know how much a nutrient is bioavailable is more important than knowing how much of it is present in a specific food or dietary supplement [19]. Indirect evidence of polyphenols absorption through the gut barrier is given by the increase in the antioxidant capacity of the plasma after the consumption of polyphenols-rich foods [20,21]. On the other hand, direct evidence on the bioavailability of phenolic compounds has been obtained by measuring their concentration in plasma and urine after the ingestion of either pure compounds or foodstuffs with a known content of the compounds of interest [22,23]. The plasma concentration of intact flavonoids rarely exceeds 1 μM, and the maintenance of a high-polyphenol concentration in plasma requires repeated ingestion [24]. Their maximum concentrations are often reached one to two hours after ingestion [25], but this is not true for polyphenols, which need to be degraded prior to absorption [26].

**Figure 2:**
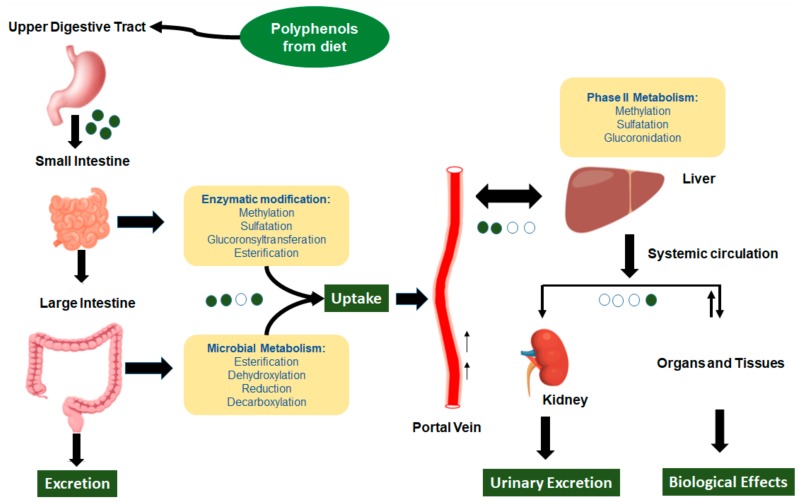
Schematic representation of the absorption and metabolism of dietary polyphenols. Green dots represent aglycon polyphenols, and white dots represent their metabolites.

There is no relation between the quantity of polyphenols in food and their bioavailability in the human body. Indeed, the limit and the absorption speed of polyphenols in the intestine are due to their chemical structure. Generally, the aglycones can be absorbed from the small intestine, but the polyphenols present in the form of esters, glycosides, or polymers cannot be absorbed in the native form. Therefore, they are hydrolyzed by intestinal enzymes, such as β-glucosidases and lactase-phlorizin hydrolase, or by the colonic microflora, and then adsorbed later (Figure 2) [27,28]. Thus, polyphenol forms that are present in the human blood and tissues are different from those present in food, making the identification of all the metabolites and the evaluation of their biological activity difficult [29,30].

The most common polyphenols in our diet are not necessarily those leading to the highest concentrations of active metabolites in target tissues; consequently, the biological properties of polyphenols greatly differ from one polyphenol to another. Indeed, polyphenols’ chemical structure is more important than their concentration, as it determines the rate and extent of absorption and the nature of metabolites circulating in the plasma. The intact glycosides of quercetin—the isoflavones daidzein and genistein—were not recovered in plasma or urine after their ingestion as pure compounds or from complex food [31,32]. An exception is represented by anthocyanins; in fact, its intact glycosides are the most representative circulating forms, but Felgines et al. also identified glucuronides and the sulfates of anthocyanins in human urine [33]. In addition, glycosylation protects resveratrol from oxidative degradation; therefore, glycosylated resveratrol is more stable, more soluble, and readily absorbed in the human gastrointestinal tract [34]. Consequently, the identification, quantification, and individual differences among polyphenols metabolites are important issues in the research for the health effects of polyphenols in humans. In fact, the colonic microflora produces specific active metabolites—for example, equol, enterolactone, and enterodiol—but the individual variability in the composition of intestinal flora leads to a different production of active metabolites, adding another degree of complexity to the understanding of the effects of polyphenols on human health [32,35].

### 2.2. Polyphenols and Cancer

Cancer is a major cause of death across the world. It is related to a group of diseases characterized by an alteration in the control of metabolism and cell growth [36]. In the continuous search for new therapies against cancer, natural polyphenols have offered some hope. Indeed, many in vitro and in vivo studies suggest that polyphenols could interfere with tumor growth and dissemination [5,37]. Polyphenols have been the subject of intense mechanistic in vitro studies, which together with epidemiological observations have led investigators to focus on the clinical properties of such compounds, applying pilot and relatively short-term clinical studies to support their cancer chemopreventive effects. In this regard, early clinical studies have been performed in healthy volunteers to explore the pharmacokinetics of polyphenolic compounds, and in individuals with premalignancies or cancer patients to define the chemopreventive and chemotherapeutic effects of polyphenols. So far, among the others, only the clinical investigations on green tea extracts have given consistent results supporting the advancement into long-term phase III clinical intervention studies to prove they chemopreventive functions in prostate cancer, leukoplakia, and premalignant cervical disease. On the other side, the clinical study of the effect of soy isoflavones and curcumin requires multiple additional trials to sustain their role in the prevention of cancer development and progression (for a review, see [38]). Altogether, the protective effect of polyphenols against cancer development remains openly debated, mainly due to a large discrepancy between mechanistic in vitro/in vivo studies and clinical observations [39,40]. The main reason for such incongruences is related to the common use, in mechanistic studies, of non-physiological concentrations of polyphenols, which could not be easily reached after food intake [39,40].

For many years, the antioxidant function of polyphenols has been considered the main mechanism of action of such compounds [3,4], and commonly associated with their ability to reduce the risk of various degenerative diseases linked to oxidative stress, including cancer [3,12,41,42]. This capacity may be explained either by the presence of reducing polyphenols and their metabolites in plasma, or by their effect on the absorption of pro-oxidative food components, such as iron [3]. The phenolic groups present in polyphenols’ structure can accept an electron to form relatively stable phenoxyl radicals, thereby disrupting chain oxidation reactions in cellular components [43]. On the other hand, polyphenols show pro-oxidant properties too, by which they could induce apoptosis and inhibit cancer growth [3,44]. Flavonoids autoxidize in aqueous medium and may form highly reactive radicals in the presence of transition metals. Moreover, polyphenols may act as substrates for peroxidase and other metalloenzymes, yielding quinone- or quinone methide-type pro-oxidant and/or alkylating products [45]. Given the high concentration of polyphenols required for these activities [4], the impact of polyphenols on cancer onset and progression appears to be related to other cellular effects besides the modulation of oxidative stress [3,4]. The antioxidant function of polyphenols has been overestimated, mainly limiting the full understanding of their functionality and their “beneficial” or “not beneficial” effect on cancer disease.

Certain polyphenols, including flavonoids, lignans, and phenolic alcohols are thought to be anticarcinogenic via a variety of mechanisms, which affect cancer cell apoptosis and the proliferation pathway [5]. Polyphenols modulate the activity of signaling pathways involved in cancer cell proliferation, including mitogen-activated protein kinase (MAPK) and phosphatidylinositide 3-kinase (PI3K) pathways [5,46]. The inhibition of the cyclic Adenosine Mono Phosphate (cAMP) response element binding protein (CREB) and/or extracellular signal-regulated kinases 1 and 2 (ERK1/2) signaling appear to be the mechanisms that are commonly impaired by certain polyphenols to induce a block in cell cycle progression [47,48,49]. The anthocyanin delphinidin possesses strong anticancer activities, inducing apoptosis and cell cycle arrest in several types of cancer, in particular suppressing the nuclear factor κ-light-chain-enhancer of activated B cells (NF-κB) pathway [50,51]. Among the flavanones, which are abundant in citrus fruits, naringenin shows anticancer effects in different cell lines. In colon cancer cells, the p38 (another member of MAP kinase family) pathway mediates the naringenin pro-apoptotic activity [52,53]. In hepatoma cells (HepG2), naringenin treatment induces mitochondrial-mediated apoptosis and cell cycle arrest through the upregulation of p53 [54].

Although several possible polyphenols mechanisms of action have been elucidated, most of the reported findings show different effects at low or high polyphenols concentrations [39,55]. Such polyphenols concentration-dependent biphasic effects might rely on their ability to interact with and modulate hormone receptors [4], sustaining an impact of these compounds on endocrine functions [3]. Given the presence of phenolic rings in both polyphenols and E2, the importance of polyphenols’ ability to impact on E2 signaling has been emphasized in different studies [10,56,57]. In addition, a large body of evidence supports a “protective” effect of dietary polyphenols in hormone-related disorders and diseases, including menopausal symptoms, osteoporosis, atherosclerosis, and cardiovascular and neurodegenerative disease [10,58], suggesting that the main biological actions of polyphenols are implemented through their modulation of ER signaling [10].

### 2.3. Polyphenols as Ligand of Estrogen Receptors

Although communally used, the term phytoestrogen is misleading to indicate phytochemical compounds, or their metabolites, which induce a biological response in mammals by combining with ERs and modulating the effect of endogenous estrogens [10,59]. Common constituents of vegetable oils and, as well of human diet, are the phytosterols or plant sterols that are produced by some plants and similar to the animal cholesterol, which is the precursor of steroid hormones. However, phytosterols are not phytoestrogens; indeed, the common plant sterols such as β-sitosterols, campesterol, and stigmasterol do not bind to human ERs, and do not activate any estrogenic activity in human or animal models [60]. 

The chemical structure of polyphenols defines their ERs binding affinity, which, despite differences among compounds, is always lower compared with the natural ligand E2 [4,57,61,62]. In addition, unlike E2, which binds with a similar affinity to both ERα and ERβ, competition-binding studies confirm that many polyphenols have different affinity for the two ERs. Among the others, genistein, daidzein, its metabolite equol, and the coumestan coumestrol show a higher affinity for ERβ than ERα [63,64,65]. Contrarily, 8-prenylnaringenin, a prenylated chalcone, has been found to be a 100-fold more potent ERα agonist, but weaker for ERβ than genistein [66]. Therefore, due to the different affinity toward ERα and ERβ, the final effect of exposure to a particular polyphenol with ERs modulation activity is mainly dependent on the target tissues/cell type and their different patterns of ERα/ERβ expression [67]. To add another dimension of complexity to this scenario, polyphenols can exert both estrogenic and antiestrogenic effects [10,56].

## 3. Estrogen Receptors: Structure and Molecular Mechanisms of Action

ERα and ERβ are ligand-activated transcription factors that mediate the cellular effect of E2 [68,69]. Although ERα and ERβ share an elevated percentage of homology (95%), they are the product of two different genes, which are located on different chromosomes [69,70]. ERs are modular proteins that are composed of six standard domains: A/B, C, D E, and F domains. Among them, the C domain, or DNA-binding domain (DBD), induces the dimerization of the receptor and allows the binding to the estrogen response elements (ERE) on DNA, the E or ligand-binding domain (LBD) occurs in the *C*-terminal region of the protein, and the hinge region (D domain) also contributes to the receptor dimerization [69,70,71,72]. In addition, two transcriptional activation (AF) regions are present: a ligand-independent domain (AF-1) at the protein *N*-terminus that corresponds to the A/B region, and a ligand-dependent one (AF-2) located at the *C*-terminus [69,70,73]. Both interact with a series of coregulators that allow the two AFs to act both in a synergic or independent way [69,73]. Mutations on both AF-1 and AF-2 can modify the ER-induced gene transcription activity and pattern by activating or inactivating the receptor, or by altering the coregulators’ binding site, and therefore altering the ER functionality [69,74].

Given their nature as ligand-activated transcription factors, classical ER activities rely on their ability to modulate the transcription of specific target genes, bind to the ERE boxes of the DNA, and recruit a series of diverse coregulators that are needed for the transcription induction [70,71,75,76,77]. When the hormone is not present, the cytoplasmic ERs are maintained inactivated through the binding with heat shock proteins (HSPs, i.e., Hsp90, Hsp70, Hsp65) and face degradation [69,70,71,75]. However, in the presence of E2, the high affinity with the hormone reverts the association with the HSPs and induces the formation of the E2–ER complex, its dimerization, and its relocation to the nucleus [69,70,71,75].

In parallel, a non-classical transcriptional mechanism also occurs where ERs regulate gene transcription without interacting directly with the ERE segments. Here, ERs bind to specific transcription factors, such as CREB or Fos and Jun proteins, which activate the activator protein 1 (AP-1) and the stimulating protein 1 (Sp1), leading to the transcriptional activation of multiple growth regulatory genes [70,71]. Furthermore, it is well known that ERs are also able to induce rapid responses through extranuclear-initiated kinase signaling cascades that can further induce cytoskeleton remodeling, cell proliferation, and cell survival mechanisms [70,78,79]. ERs are located at the plasma membrane, in the caveolae [80] or in different other membrane raft structures [69,70,81]. The ER *S*-palmitoylation, which occurs within the E domain, is the main determinant of the ERα and ERβ membrane localization, caveolin-1 interaction, and the activation of E2 rapid signaling [70,82,83]. E2, via the membrane-localized ERs, quickly (from seconds to minutes) induces several signal transduction pathways, including but not limited to, phospholipase C (PLC)/protein kinase C (PKC), p38/MAPK, and PI3K pathways, leading to increased levels of second messengers [70,72,84]. On the other hand, the E2–ERα complex can associate with different growth factor receptors (epidermal growth factor receptor—EGFR—or insulin-like growth factor 1 receptor—IGF1-R) on specific docking sites, evidencing the cross-talk interaction between E2 and growth factor signaling [85]. 

Recently, another receptor belonging to the wide class of G-coupled receptors, named GPER, has been thought to mediate the rapid extranuclear effect of E2 [86,87]. However, so far, the actual role of such a receptor in E2 signaling and effects is still controversial and mostly debated [88,89]; so, GPER signaling will not be analyzed here.

## 4. ERs-Based Polyphenol Effects on Cancer Hallmarks

ERs are widely distributed in the organism, showing a different and sometimes overlapped expression pattern [69,72,82]. ERα is mainly found in breast, uterus, prostate, liver, brain, bones, and adipose tissue, and ERβ is mainly found on the ovaries, testes, prostate, colon, lung, spleen, thymus, bone marrow, salivary gland, and vascular epithelium [72]. Consequently, E2 has a wide range of target tissues; thus, it is not surprising that E2 is involved in the insurgence of multiple diseases, including different types of cancers such as breast, ovarian, colorectal, endometrial, and prostate cancers [90]. Despite the beneficial physiological actions of normal levels of E2, alteration of the E2 signaling or the imbalance of the ERs’ distribution has been correlated with cancer progression, as well as with the response to the treatments and prognosis [91,92]. In particular, the alteration of the ERα/ERβ ratio in the affected tissues is one of the main reasons of the variability of estrogen-dependent cancer biology [93,94,95]. For example, more than 70% of diagnosed breast cancers are ER-positive [96], and modified ER signaling might lead to an aberrant cellular proliferation and survival, prompting the progression and development of the breast cancer. Consistently, invasive breast cancer shows an elevated expression of ERα present on more than the 75% of cells, while ERβ expression has been demonstrated to decrease in respect to the normal condition [74]. On the contrary, in lung and digestive cancers (colorectal, esophageal), where ERβ is the main expressed ER subtype, E2 displays protective anti-cancer function, as suggested by epidemiological data [97,98]. The restoration of ERβ expression in ovarian cancer cells [99] and breast cancer cells [100] results in an increasing apoptosis and a strong inhibition of proliferation and invasion, further supporting the role of ERβ as a tumor suppressor. 

Beyond the polyphenol metabolism, bioavailability, and time of exposure, several factors related to their (anti) estrogenicity on ERα and/or ERβ define the complexity of polyphenols’ effects on cancer cells and sustain the need for a deep understanding of the molecular pathways involved in the ER-mediated effects of polyphenols on the cancer initiation, progression, and development that impact on the overall disease process. In the following sections, the ER-mediated molecular mechanisms of polyphenol impact on the physio/pathological processes of cancer cells, including their growth, survival, and migration, will be discussed.

### 4.1. Cancer Cell Proliferation

In the last decades, the mechanism activated by E2 to induce cell proliferation has been the object of extensive studies in different tissues [101,102,103,104,105,106]. Despite E2-related effects occurring, as reported before, through the direct regulation of genes transcription by nuclear localized ERα and ERβ or triggering rapid signaling by membrane-associated receptors, many compelling lines state that E2 proliferative functions are mainly mediated by the ERα extranuclear activities [105,107,108]. In human ERα-positive breast cancer cells, such as MCF-7, E2 treatment leads to the cell cycle progression, inducing the activation of PI3K through the binding of ERα, but not ERβ, to the PI3K regulatory subunit p85 [103,109]. The absence of any E2 proliferative effects on ER-negative MDA-MB-231 cells further supports that the E2 activation of PI3K/protein kinase B (AKT or PKB) pathway is dependent on ERα expression [109]. Consistent with this, the same PI3K/AKT pathway was found to be pivotal in the E2-dependent proliferative effect on chondrocytes [110], as well in endometrial cancer cells [111]. Together with other pathways (i.e., ERK/MAPK), it was also found in human hepatoma cells (HepG2), where the inhibition of such pathways completely prevents the hormone-induced DNA synthesis and cell cycle progression [56,107,108]. On the contrary, cell and animal-based findings indicate that ERβ mediates E2 anti-proliferative functions, antagonizing, in many ways, ERα activities [56,68,82]. Indeed, in transfected MCF-7 cells, ERβ inhibits E2-dependent proliferation and prevents tumor formation in a mouse xenograft model [112]. In addition, E2 significantly reduces the prostate cancer cell proliferation rate through the ERβ-dependent increase of the cyclin-dependent kinase inhibitor [113].

Dietary polyphenols may potentially modify cancer cell cycle progress, affecting or blocking ER-dependent cancer progression. Indeed, given the distinct pro- and anti-cancerous effects mediated by ERα and ERβ, respectively, the balance of ERα and ERβ expression together with the specific function of polyphenols on the different subtypes represent the main factor that defines the effects of such compounds on cancer progression. 

We identified that both naringenin and quercetin impair, even in the presence of E2, the ERα-activated rapid signaling (i.e., receptor palmitoylation*,* ERK/MAPK and PI3K/AKT activation) that is important for the cyclin D1 expression, and consequently for cell cycle progression, without affecting the transcriptional effect of activated ERα [52,114]. Conversely, studies on the effects of resveratrol and genistein on cancer cell proliferation have generated conflicted results. Indeed, low concentrations of resveratrol and genistein have been found to induce cell growth in ER-sensitive breast cancer cells [115,116,117]. Other independent studies affirm that genistein induced the proliferation of MCF-7 cells and S-phase entry through the trans-activation of ERα and a delayed and prolonged activation of ERK1/2 [118]. In vivo studies further support such a proliferative function of genistein, which stimulates the growth of E2-dependent mammary tumors in ovariectomized rats [119], as well the proliferation of implanted MCF-7 breast cancer cells in xenografts mice [120]. Similarly, the proliferative effect of a low concentration of daidzein and its metabolite equol on MCF-7 cells has been found to be dependent on ERα activation [121] (Table 1). 

On the other side, high concentrations of resveratrol, genistein, daidzein, and equol (i.e., micromolar range) suppress the proliferation of E2-sensitive cells, including breast and ovarian cancer cells, suggesting the concentration-dependent biphasic effect of such compounds [57,117,122,123,124], and raising questions about the physio/pathological effects of these high concentrations, that usually do not occur on human tissue after dietary consumption. 

In contrast, consistent with the above reported ERβ anti-proliferative and antagonistic effects on ERα [56,68,82], soy isoflavones (genistein, daidzein, glycitein) suppress colon carcinoma cell growth by decreasing, via ERβ, the mitogenic signaling pathways ERK1/2 and PI3K/Akt, and the expression of proliferating cell nuclear antigen (PCNA) and NF-κB [125]. Similarly, both daidzein and equol reduce proliferation in an ERβ-dependent way when the receptor was transfected into cervices carcinoma (HeLa) cells [126].

As a whole, the effect of polyphenols on cancer cell proliferation may differ depending on the ratio of ERα and ERβ expression and their different selectivity and concentration, displaying the necessity of a complete picture of dietary polyphenols functions that takes into account their effects at multiple levels (Table 1).

### 4.2. Cancer Cell Escape from Apoptosis

Besides the regulation of proliferation, E2 could affect cancer progression and development through the direct modulation of cell survival [70,81,82]. Indeed, in line with the pro-cancerogenic effect of ERα signaling, the E2/ERα-activated ERK/MAPK and PI3K/AKT pathways deserve attention related to the E2 action as a pro-survival agent [70,79]. In fact, these are involved in the induction of the expression of the anti-apoptotic protein Bcl-2, the inactivation of the pro-apoptotic p38/MAPK signaling, and the inhibition of the caspase-3-dependent pro-apoptotic pathway in E2-sensitive cancer cells [56,82,138]. In addition, we recently found the monomeric globin neuroglobin (Ngb) as one of the key compensatory proteins that is upregulated and reallocated to mitochondria by E2 via the ERα/PI3K/AKT pathway. Ngb mediates the hormone-dependent anti-apoptotic and pro-survival effect in sensitive breast cancer cells [139,140]. On the contrary, E2 triggers, through the ERβ subtype, a pro-apoptotic effect in prostate cancer cells via the tumor necrosis factor alpha (TNFα) signaling [141], and in colon cancer cells through activating the p38/MAPK pathway [82,129], confirming the strong dependence of the effect of E2 on the balance between the expression and signaling activated by ERα or ERβ [70,79,95]. 

The ability to induce programmed cell death in different cell lines including breast, lung, leukemia, colorectal, and prostate cancer cells [142,143,144,145,146,147] has been ascribed to different dietary polyphenols. Also, regarding the effect on cancer cell growth, the apoptotic pathway appears to be differently affected by polyphenols depending on their type and concentration, showing a biphasic effect [55,148]. As previously mentioned, at molecular levels, a number of key elements of the cellular apoptotic signaling pathway have been involved in the polyphenol-dependent regulation of apoptosis [55,148,149]. Polyphenol ability to induce apoptotic cell death is associated with a decreased expression of the anti-apoptotic protein Bcl-2, which is paralleled with the increase of pro-apoptotic proteins such as BAX, Bak, and Bim, as well with the upregulation of p53, p21, and p27 in several type of cancer cells (for a review, see [46]. In addition, polyphenols trigger the extrinsic apoptotic pathway, activating the Jun *N*-terminal kinase (JNK)/p38 pathway in human gastric carcinoma cells [150]. So far, the role and involvement of ERα and/or ERβ in the polyphenol-dependent modulation of cancer cell apoptosis has been only partially investigated. However, the different agonist and/or antagonist effects of many polyphenols compounds on ERα and ERβ, and the relative expression of each ERs subtypes, which define the final cellular response, can explain the controversial pro and anti-apoptotic functions of polyphenols that are sometimes reported in different cancer cells. 

Quercetin and naringenin, at nutritionally relevant concentrations, decouple ERα-mediated rapid signaling, preventing the anti-apoptotic and proliferative ERK/MAPK and PI3K/AKT pathway activation, and sustaining the persistent phosphorylation of p38/MAPK, and in turn, the blockage of cell cycle progression and the induction of the pro-apoptotic cascade [129,151]. Thus, both naringenin and quercetin influence cancer cell proliferation and survival through acting as partial antagonists of ERα-activated rapid signals [56,129,151]. On the contrary, both flavonoids behave as E2-mimetic agents in the presence of ERβ by activating the p38/MAPK and the downstream pro-apoptotic pathway, indicating the pivotal role of both ER subtypes’ expression patterns in the target cell/tissue and the differential agonist or partial antagonistic effects of these compounds in the definition of their anti-carcinogenic potential (Table 1) [130,152].

Sakamoto et al. further sustained that the effects of polyphenols on the survival of cancer cells might rely on ER-dependent mechanisms. They showed that resveratrol is able to induce pro-apoptotic effects through reducing the Bcl-2/BAX ratio at least in part via an ERα-mediated mechanism, although the exact mechanism by which it occurs was not completely elucidated [55]. Consistently, resveratrol induces apoptosis more in the E2-sensitive breast cancer cells MCF-7 than in E2-insensitive cells such as MDA-MB-231, suggesting the involvement of ERs [153]. Many lines of compelling data indicate the role of resveratrol in sensitizing cancer cells to the action of several anti-cancer drugs [154,155,156], including paclitaxel, which is an antineoplastic agent that is commonly used in breast cancer clinical practice [157,158]. Our recent findings indicate [159] that resveratrol sensitizes breast cancer to paclitaxel by downregulating Ngb through its action on ERα-dependent activity [128]. Similarly, naringenin also increases the susceptibility of breast cancer cells to paclitaxel by interfering with the E2-activated pathway that is involved in Ngb upregulation (Cipolletti personal comunication). As a whole, both resveratrol and naringenin promote cancer cell death affecting the E2/ERα/Ngb pathway. Remarkably, our data further indicate that resveratrol and naringenin behave as full or partial E2 antagonists in ERα-positive and ERβ-negative cells. Indeed, both phytochemicals increase Ngb levels, and the consequent cell survival, in neuron-derived cells where the ERβ subtype prevails (Table 1) [128]. 

Furthermore, many authors have suggested that the conflicting results concerning the effect of polyphenols on cancer cells survival rely on the endogenous E2 concentrations, remarking on the complex mode of action of many polyphenols [160,161,162]. Indeed, it has been found that low concentrations (< 1 mmol/L) of genistein, equol, and coumestrol induce the growth of MCF-7 breast cancer cells adapted to an enriched E2-enviroment, while inducing cell apoptosis in long-term E2-deprived MCF-7 through an ERα-dependent mechanism [160].

### 4.3. Cancer Migration and Metastasis

Activating local cell invasion and distant metastasis represents another important hallmark of cancer that mainly reflects the progression of epithelial-derived carcinomas to a higher grade of malignancy [163]. Cell migration is a critical process for cancer cell spread, invasion, and distant metastasis, and it results from a dynamic remodeling of intracellular cytoskeleton and focal adhesion sites in parallel with the disruption of focal contacts, the formation of specialized structures, such as lamellipodia and filopodia, and the definition of new focal points for cell motility [164,165,166]. Several extracellular factors (i.e., growth factors) deeply influence cell migration through the activation of intracellular pathways [164]. Among them, sex steroid hormones arise as key fundamental regulators of cell morphology and motility in different normal and cancer cells [167,168].

ERα rapid extranuclear signaling appears to be crucial in the E2-dependent regulation of cancer cell cytoskeleton remodeling, migration, and invasion [167,169]. Among the others, the ERα-dependent activation of the Rous sarcoma virus (Src) tyrosine kinase kinase, MAPK, AKT, and PKC pathways are implicated in the E2-mediated regulation of cell motility [164,167,168]. In particular, integrin-linked kinase (ILK) [164], ER regulator PELP-1 protein [167,170], and the formation of a multiprotein complex between ERα, Src, PI3K, and the actin-regulator focal adhesion kinase (FAK) [168] have been suggested as crucial players in the invasion and motility of breast cancer. In addition, the cross-talk between ERα and different other membrane receptors, including EGFR, IGF-R, and epidermal growth factor receptor 2 (HER2) is involved as a mechanism of regulation of cytoskeleton reorganization [167,171]. To confirm further the role of ERs signaling in cell migration, in ERα-positive breast cancers around 60–70% of distant metastasis retain the expression of the receptor. In addition, the ERα-mediated signaling appears to be correlated with bone and lung metastasis, while ERβ expression promotes anti-migratory and anti-invasion cellular response [167]. As a whole, several findings support the role of ER signaling in modulating metastasis and cancer invasion, opening the consequent possibility that the polyphenols can act on such mechanisms via the regulation of ER activity.

Data regarding the effects of polyphenols on cancer cell migration are still controversial and the molecular mechanisms that define them are not completely elucidated. In vitro assays indicate that genistein and daidzein diminish the migration of breast cancer cells through the inhibition of the NF-κB pathway [134]. In addition, genistein treatment reduces cell invasion in prostate cancer [172] as well in hepatocarcinoma cells [173]. An increased metastatic capability in cell migration and invasion is strictly linked to the cell epithelial–mesenchymal transition (EMT) process, which represents a critical step in cancer progression, as it is responsible for profound phenotypic and morphological change that promote cell motility [134,174]. Among the others, resveratrol, genistein, and the flavonoid kaempferol inhibit EMT transition, favoring the expression of the epithelial phenotype-related gene (i.e., e-cadherin) and reducing the expression of mesenchymal phenotype related-ones (i.e., N-cadherin, Snail, Slug, and vimentin), which results in the reduction of cell migration (for a review, see [134]). Thus, many lines of compelling data suggest the anti-migratory effect of polyphenols. Nevertheless, in melanoma cells, only an extremely high concentration of genistein (~100 µM) is able to prevent cancer migration via inhibiting the FAK/paxillin and MAPK pathways, which are activated, on the contrary, by a lower genistein concentration, leading to an increased cell invasion and migration [175]. Furthermore, in an in vivo model of breast cancer, genistein, daidzein, or equol at blood concentrations similar to those observed in humans after a soy-based diet increase the lung metastasis, suggesting the pro-cancerous effect of such compounds acting on cell invasion and migration [136]. Thus, skepticism on polyphenol anti-migratory effects have arisen in particular due to their reported concentration-dependent biphasic effects [134]. This dual functional effect of polyphenols on cancer cell migration appears to be conceivable with the ER-mediated effect of these compounds, in particular at low and more attainable concentrations. Indeed, the genistein and daidzein effects that are observed on breast cancer metastasis could rely on their agonist effect on ERα, which is consistent with the reported proliferative function of such compounds through the activation of this ER subtype [118,121].

On the contrary, in ovarian cancer cells, the inhibitory effect of genistein and daidzein has been found to suppress cell migration through the downregulation of different migration-related pathways (i.e., FAK, PI3K/AKT), which implies a role in ERβ activation (Table 1) [131,134]. Accordingly, genistein inhibits the tumor growth and distant metastasis of mice colorectal tumor [133], where the ERβ-mediated functions prevail (for a review, see [176]), and, through an ER-mediated mechanism, genistein favors cell adhesion, impairing the migration of DU145 and PC3 prostate cancer cells, which express only ERβ subtypes [132]. Thus, given the anti-carcinogenic role of ERβ-mediated signaling [82,134], the polyphenol anti-migratory effect might rely on the activation of such ER subtype. On the other hand, kaempferol suppresses the EMT transition and the metastatic behavior of breast cancer MCF-7 cells induced by both E2 and estrogen mimetic compounds through a mechanism that suggests a kaempferol antagonistic effect on ERα (Table 1) [137]. Alternatively, Shang et al. suggested that the flavonoid baicalein inhibits the E2-mediated migratory effect in cancer cells by interfering with the hormone-dependent activation of GPER [177].

Altogether, many of polyphenol effects on cell migration, at concentrations that are more conceivable with those attainable after food ingestion, might rely on the differential effects on ER signaling, acting as pro- or anti-migratory compounds based on their agonistic/antagonistic or mixed functions, and the relative balance of ERα and ERβ expression in target tissues (Table 1). Nevertheless, only a few works have aimed at evaluating the direct involvement of the ER-activated cellular response in polyphenol effect on cancer migration, and future studies will need to shed light on their actual effect on this cancer hallmark.

## 5. Conclusions and Perspective

The prevention of cancer through dietary interventions has become an important issue in recent years [46]. There is a broad consensus that a high-level daily intake of fruits and vegetables will help prevent the occurrence and progression of cancer, particularly related to their high polyphenols content [3,46]. Nevertheless, although some studies have confirmed the association between the consumption of fruits and vegetables and the reduction of cancer incidence [178,179], other epidemiological reports asserted that no, or very little, relationship is present between fruit and vegetable consumption and overall cancer risk [142,180,181,182]. Consistently, several concerns about the beneficial effects of dietary polyphenols on cancer risk have been also arisen from animal and in vitro studies [8,183,184]. Such incongruences rely on different issues, mainly including: the (i) generalization of polyphenol effect on “cancer” without taking the considerable diversity of their chemical structures and metabolites into account, (ii) the cell type or context where the polyphenol effect has been analyzed, and (iii) the concentration of polyphenols that was used for the assays [46].

Beyond the long-studied effects of polyphenols on the modulation of cell oxidative status, several mechanisms of action have been identified to explain the anti-cancer effects of natural polyphenols, including anti-inflammatory activities and the modulation of signaling pathways, which are associated with cell survival, proliferation, differentiation, migration, angiogenesis, hormone activities, and detoxification enzymes [185,186].

**Figure 3:**
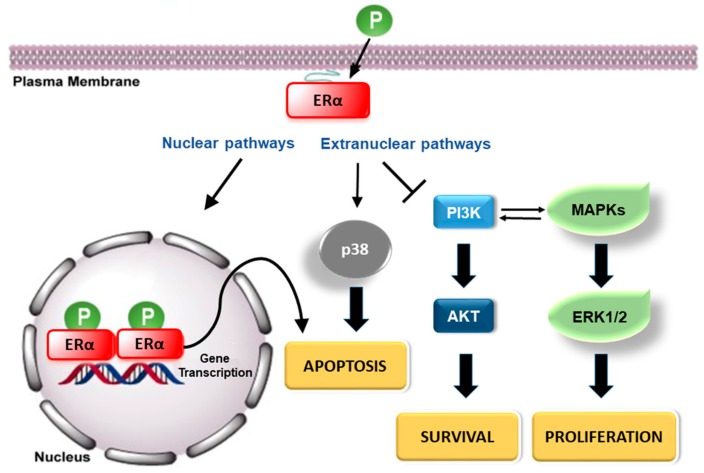
Schematic representation of the 17β-estradiol (E2) antagonist mechanisms triggered by polyphenols (P) that drive cancer cells to apoptosis in the presence of ERα.

Among the other mechanisms proposed, the ability of some polyphenols to bind to ERs in vitro, and, consequently modulate the E2-signaling pathway, has become the crucial process on which the diverse and sometimes opposing functions of polyphenols might rely. Polyphenols can act as E2 agonists and antagonists at the same time, usually in an organ-dependent manner, resulting in mixed agonistic/antagonistic properties (selectivity) depending on the receptor content of specific tissues (ERα and ERβ populations) and the concentration of the endogenous estrogens [10]. Figure 3 shows a schematic example of E2 antagonist mechanisms triggered by polyphenols that drive cancer cells to apoptosis in the presence of ERα. In addition, based on findings about the polyphenol metabolism and bioavailability, metabolites, more so than the native form of polyphenols that is found in food, are often the active compounds that affect cell functionality, although they have been not sufficiently tested in in vitro and in vivo studies [3,13].

In the light of different degrees of estrogenicity, the ER selectivity of different polyphenols compounds [10], and given the different and sometime opposing functions mediated by ERα and ERβ on cancer cells [82], the safety of polyphenols is still a concern for human health. Therefore, it now appears clear how the assessment of estrogen-like effects and anti-tumor functions of any singular dietary polyphenols are crucial to fully understanding the role of polyphenols in cancer therapy, as well their safe use [10,55,187]. Thus, further investigations into the effects of dietary polyphenols through the modulation of ER pathways are warranted, as well a re-evaluation of all of the complex experimental studies and clinical approaches to define safer nutritional recommendations regarding the impact of dietary polyphenols on human health. In this regard, focusing on specific dietary polyphenols and their ER-mediated molecular mechanisms, coupled with advanced delivery systems (i.e., nanoparticles) to enhance the bioavailability of selected compounds, may open new avenues in the identification of healthy natural compounds and their possible use in therapeutic interventions.

## Figures and Tables

**Table 1:** Summary of known effects of dietary polyphenols on cancer hallmarks through estrogen receptor subtype (ERα or ERβ)-mediated molecular mechanisms.

Polyphenol Compounds	ERα	ERα-Dependent Functions	ERβ	ERβ-Dependent Functions	References
**Naringenin Quercetin**	Antagonize ERα rapid signaling, inducing the persistent activation of p38, the inhibition of AKT and ERK1/2 E2-dependent activation. No effect on ERα transcriptional mechanism.	Inhibit proliferation in ERα-expressing cancer cells and induce pro-apoptotic cascade. Impair the E2-dependent upregulation of Ngb and sensitize breast cancer cells to apoptotic effect of paclitaxel.	Mimic E2 effect activating p38/MAPK pathway	Induction of ERβ mediated pro-apoptotic pathway	[52,114,127,128,129,130]
**Genistein**	Agonistic effect. Trans-activation of ERα inducing the persistent activation of ERK1/2.	Induction of MCF-7 cell proliferation in vitro and implanted in xenograft mice; stimulation of E2-dependent mammary tumors’growth. Inhibits cell growth and induces apoptosis in long-term estrogen deprived MCF7	E2 mimetic effects. Suppression of the ERK1/2, PI3K/AKT activation and PCNA and NFkB expression. Downregulation of migration-related pathways such as the FAK, PI3K/AKT and TGFβ pathways.	Suppression of cancer cell growth. Inhibition of migration in ovarian and prostate cancer cells and reduction of cancer metastasis in colorectal cancer.	[118,119,125,131,132,133]
**Daidzein Equol**	Agonist	Induce in vitro MCF-7 cell proliferation Increase lung metastasis in in vivo model of breast cancer.	Agonist	Inhibit cancer cells proliferation. Suppress ovarian cancer cell migration.	[125,126,134,135,136]
**Resveratrol**	Full antagonist of ERα rapid and transcriptional mechanisms	Induce pro-apoptotic effects reducing the Bcl-2/BAX ratio. Downregulates Ngb intracellular content and sensitizes breast cancer cells to the paclitaxel pro-apoptotic effect.	Agonist	Increases Ngb levels and cell-survival in neuron-derived cells	[55,128]
**Kaempferol**	Antagonist	Suppresses EMT transition and metastic behavior of MCF-7 induced by endogenous E2 or estrogen mimetic compounds.	ND	ND	[137]

EMT. Epithelial–mesenchymal transition; Ngb. Neuroglobin. For further elucidation see the text.
